# Evaluating the role of quantitative pupillometry in chronic subdural hematoma: A pilot study

**DOI:** 10.1016/j.bas.2025.104391

**Published:** 2025-08-13

**Authors:** Lukas Klein, Christopher Beynon, Daniel Kühlwein, Sandro M. Krieg, Alexander Younsi, Pavlina Lenga

**Affiliations:** a Department of Neurosurgery, Heidelberg University Hospital, Heidelberg, Germany; b Medical Faculty of Heidelberg University, Heidelberg, Germany; c Department of Neurosurgery, Mannheim University Hospital, Mannheim, Germany

**Keywords:** Chronic subdural hematoma, Pupillometry, Intracranial pressure, Brain atrophy, Computed tomography

## Abstract

**Introduction:**

As the incidence of chronic subdural hematoma (cSDH) increases with an aging population, identifying noninvasive methods for early detection and monitoring is crucial. Brain atrophy in older adults creates additional intracranial reserve, allowing large hematomas to accumulate without significantly elevating intracranial pressure (ICP). We investigated whether quantitative pupillometry (QP) could detect subtle pressure changes or neurological compromise in these patients.

**Methods:**

This prospective study included 26 elderly cSDH patients between 65 and 97 years of age undergoing standard cranial computed tomography (cCT) and surgical burr-hole hematoma evacuation at our institution. Pupillometry assessments were conducted prospectively using the automated NPi 200® Pupillometer and the Neurological Pupil Index (NPi) was measured pre- and post-operatively. Clinical and radiological parameters including the initial Glasgow Coma Scale (GSC) score, hematoma volume, residual cavity area (RCA), and the Evans index were obtained and all parameters were statistically compared. In addition, correlation analyses were performed (p < 0.05 was considered significant).

**Results:**

The median hematoma volume was 132.5 ml (IQR 82.2 ml), with a median RCA of 4.4 mm (IQR 6.2 mm), and a median Evans index of 0.5 (IQR 0.1). Preoperatively, the mean NPI was 4.6 (SD 0.9) in the right eye and 4.5 (SD 0.5) in the left eye. No statistically significant differences were observed when directly comparing NPI, GCS, hematoma volume, RCA, Evans index, and the presence of motor deficits to one another. However, RCA showed significant positive correlations with age (rs = 0.3; p = 0.026), Evans index (rs = 0.5; p = 0.003), and hematoma volume (rs = 0.14; p = 0.043). There was also a trend toward a negative correlation between RCA and GCS (rs = −0.7; p = 0.053), as well as between hematoma volume and left-eye NPI (rs = −0.3; p = 0.058).

**Conclusions:**

In this cohort of older cSDH patients, pupillometry did not reveal any abnormal responses, suggesting that the intracranial reserve created by brain atrophy may prevent the development of elevated ICP—despite large hematoma volumes. Research is warranted to identify additional biomarkers or strategies to improve early cSDH-detection and management.

## Highlights

• This prospective pilot study is the first to evaluate the utility of quantitative pupillometry (QP) specifically in elderly patients undergoing surgical drainage for chronic subdural hematoma (cSDH).
•Despite substantial hematoma volumes, pupillometry results remained within normal limits, underscoring the complex influence of age-related brain atrophy on intracranial pressure dynamics.
•Our findings emphasize that older adults with significant cortical atrophy can harbor large subdural collections without demonstrating pupillary changes, reinforcing cranial CT as the indispensable diagnostic standard.

## 1 Introduction

Chronic subdural hematoma has become increasingly common, partly because the population is aging and partly due to the more widespread use of anticoagulant and antiplatelet therapies (Asghar et al., 2002; Adhiyaman et al., 2017; Gaist et al., 2017). Typically affecting older adults, cSDH develops over weeks to months, often triggered by minor head trauma and facilitated by age-related brain atrophy, which enhances intracranial compliance and augments the potential space for blood accumulation (Trotter, 1914; Yamashima and Friede, 1984; Lee, 2016). Despite the initial bleeding commonly arising from bridging veins at the dura-arachnoid interface, the pathophysiology is more complex than a simple venous rupture. Chronic inflammatory processes, neomembrane formation, and angiogenesis within the hematoma cavity drive sustained expansion and can produce a spectrum of clinical presentations ranging from subtle cognitive and behavioral changes to overt neurological deficits (Nagahori et al., 1993; Gandhoke et al., 2013; Nayil et al., 2012).

Diagnosis of cSDH classically relies on cranial computed tomography (CT), which provides detailed information on hematoma size, density, and related mass effect (Mori and Maeda, 2001; Macdonald and Winn, 2017). However, early detection in subclinical or resource-limited scenarios remains challenging, as patients may present with non-specific symptoms and normal intracranial pressure (ICP) for a prolonged period. Identifying an adjunctive diagnostic tool that can indicate intracranial dynamics without immediate imaging availability has thus drawn attention to noninvasive neuromonitoring techniques.

Quantitative pupillometry, which has been explored in other acute neurological settings, offers a standardized assessment of pupillary reactivity potentially sensitive to ICP changes (Chen et al., 2011; Jahns, 2019; Lussier et al., 2019; Meeker et al., 2005; Olson and Fishel, 2016). Yet, the specific physiological characteristics of cSDH, including gradual hematoma enlargement often without pronounced ICP elevation, raise critical questions about the usefulness of pupillometry in this context (Nouri et al., 2021). The present study examines whether QP can serve as a meaningful complementary diagnostic modality in patients with cSDH, particularly in the older population characterized by substantial intracranial reserve and limited early pressure shifts. In doing so, it seeks to clarify if pupillometry provides any added clinical value or if these pathophysiological conditions inherently limit its diagnostic yield. Our study sought to systematically evaluate the diagnostic utility of quantitative pupillometry in the context of cSDH, examining whether this approach could enhance early recognition, complement existing imaging modalities, and ultimately improve clinical decision-making for this vulnerable patient population.

## 2 Methods

### 2.1 Study design and population

This prospective study included elderly patients aged ≥65 years presenting with cSDH that was confirmed by cCT and surgically treated via trphination between December 2023 and October 2024 at our neurosurgical department.

Patients were excluded if they had any pre-existing severe ocular conditions (e.g., advanced glaucoma or dense cataracts) that could interfere with pupillometry, were unable to cooperate with the examination, or were receiving deep sedation or muscle relaxants that might affect pupillary reactivity. There were no exclusion criteria concerning previous neurosurgical cranial procedures.

The study was conducted in accordance with the Declaration of Helsinki and approved by the local ethics committee of our institute (approval number S518/2023).

### 2.2 Pupillometry

Quantitative pupillometry was performed preoperatively at the patient's bedside using an automated handheld pupillometer. The Neurological Pupil Index (NPI) was measured for each eye separately. Measurements were conducted under existing light conditions on the ward to avoid disrupting clinical routines. Trained staff performed the measurements according to the manufacturer's instructions to ensure standardized data collection. NPI values below 4.0 were considered pathological, indicating potential neurological compromise, while values of 4.0 and above were classified as normal.

### 2.3 Clinical and radiological data

Demographic data such as age and gender, as well as the neurological status assessed by the GCS and neurological deficits upon admission, and discharge were collected for all patients. Radiological parameters were obtained from the preoperative cCT which was typically performed upon admission.

### 2.4 Management and follow-up

All patients underwent surgical treatment via burr-hole hematoma evacuation and insertion of a subdural drain. The burr hole was placed either over the tuber parietale or the frontal region, depending on the surgeon's preference and the individual patient's imaging findings. Drains were removed after 24–48 h and patients mobilized thereafter. Postoperative monitoring included the documentation of neurological changes, complications, and potential cSDH recurrence as indicated by clinical deterioration or follow-up imaging.

QP was repeated postoperatively at patient's bedside under the same conditions as preoperatively and by the same staff.

The decision to proceed with surgical intervention was based on current clinical guidelines and standard neurosurgical practice, considering symptom severity, imaging findings, and patient comorbidities. Perioperative management, including the use of drains and postoperative imaging intervals, followed institutional protocols.

### 2.5 Radiological parameters

#### 2.5.1 Evans index (EI)

The Evans index was used to estimate intracranial atrophy by quantifying ventricular enlargement. Using an axial CT slice through the frontal horns, we measured the maximal width of these ventricles and divided it by the maximal internal skull diameter. A value above approximately 0.3 suggests ventriculomegaly (Evans, 1942). The Evans index was subsequently computed as: EI = Maximal Frontal Horn Width/Maximal Internal Skull Diameter.

#### 2.5.2 Calculation of the relative cortical atrophy index (RCA)

The RCA was additionally used to quantify the degree of cortical atrophy in each patient (Evans, 1942). The calculation was performed using the following formula:

RCA=(ICxFIxSW)/SkullDiameter

where.

- **IC** = Width of the insular cisterns (mm)
- **FI** = Width of the anterior part of the longitudinal cerebral fissure (mm)
- **SW** = Greatest width of the cerebral sulci at the skull vault (mm)
- **Skull Diameter** = Measured in millimeters on axial cCT images

All measurements were obtained using standardized anatomical landmarks and were independently evaluated by two experienced neurosurgeons.

### 2.6 Statistical analysis

All clinical, radiological, and pupillometry data were prospectively recorded in a secure electronic database. Statistical analysis was performed using IBM SPSS Statistics for Windows, Version 29.0 (IBM Corp., Armonk, NY). The distribution of data was assessed using the Kolmogorov-Smirnov test for normality. For normally distributed data, mean and standard deviation (SD) were reported; for non-normally distributed data, median and interquartile range (IQR) were used. Categorical variables were presented as absolute numbers and percentages. **C**orrelations between continuous variables were analyzed using Spearman's rank correlation coefficient. A p-value of <0.05 was considered statistically significant.

## 3 Results

### 3.1 Demographic and clinical characteristics

A total of 26 patients with cSDH were included. The mean age was 75.8 years (range 65–97), with 19 males (73.1 %) and 7 females (26.9 %). Motor deficits were present in 10 patients (38.5 %), and the median GCS was 14.0 (IQR 1.2), as shown in Table 1.

**Table 1:** Baseline characteristics.

N	26
Sex (n,%)	19 (73.1)
Male	7 (26.9)
Female	
Age (mean, SD, years)	75.8 (65–97)
cSDH (n, %)	
Left-sided	16 (61.5)
Right-sided	6 (23.1)
Bilateral	4 (15.4)
Motor deficit (n, %)	10 (38.5)
GCS (median, IQR)	14.0 (1.2)
Hematoma Volume (median, IQR)	132.5 (82.2)
RCA (median, IQR)	4.4 (6.2)
Evans Index (median, IQR)	0.5 (0.1)
Preoperative findings of QP	
NPI right eye, (mean, SD)	4.6 (0.9)
NPI left eye, (mean, SD)	4.5 (0.5)
NPI total (mean, SD)	4.4 (0.5)

cSDH: chronic subdural hematoma; GCS: Glagow Coma Scale; SD: standard deviation, IQR: interquartile range; NPI: Neurological Pupil Index; QP: quantitative pupillometry; RCA: residual cavity area.

### 3.2 Radiological measurements

The median hematoma volume was 132.5 ml (IQR 82.2 ml), the median RCA was 4.4 mm (IQR 6.2 mm), and the median EI was 0.5 (IQR 0.1).

### 3.3 Pupillometry findings

Preoperatively, the mean NPI was 4.6 (SD 0.9) in the right eye and 4.5 (SD 0.5) in the left eye, resulting in an overall mean NPI of 4.4 (SD 0.5), as displayed in Table 1.

### 3.4 Correlation analysis

**Fig. 1:**
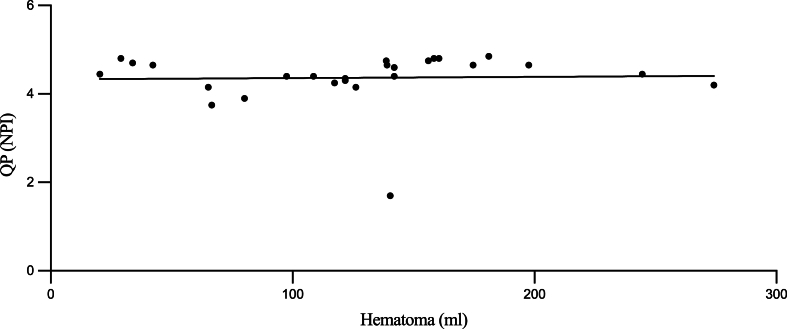
Association Between Mean Neurological Pupil Index (NPI) and Hematoma Volume A scatterplot displaying the Spearman correlation between preoperative mean NPI values (mean of left and right eye NPI) on the y-axis and hematoma volume (in milliliters) on the x-axis for 26 patients with chronic subdural hematoma. Each data point represents one patient's measured values. A nonparametric (Spearman) correlation test was used to assess the strength and direction of the association.

No significant differences were identified when comparing NPI, GCS, hematoma volume, RCA, EI, and motor deficits with each other. Significant positive correlations were noted between RCA and age (rs = 0.3; p = 0.026), RCA and EI (rs = 0.5; p = 0.003), and RCA and hematoma volume (rs = 0.14; p = 0.043). A trend toward significance was observed for negative correlations between RCA and GCS (rs = −0.7; p = 0.053) as well as between hematoma volume and left-eye NPI (rs = −0.3; p = 0.058), as depicted in Table 2. The relationship between preoperative mean NPI values (averaged across left and right eyes) and hematoma volume is further illustrated in Fig. 1. The scatterplot demonstrates a nonparametric Spearman correlation, where each data point represents an individual patient's measured values.

**Table 2:** Correlation analysis between RCA and selected demographic/radiological parameters.

		Spearman's R	p-Value
RCA	Age	0.3	**0.026**
RCA	Evans-Index	0.5	**0.003**
RCA	Volume hematoma	0.14	**0.043**

RCA: residual cavity area.

## 4 Discussion

In the diagnosis of cSDH, cCT is considered the gold standard, as it provides high sensitivity, specificity, and detailed assessments of hematoma size, location, and associated intracranial changes. Given the growing number of elderly patients with cSDH and the clinical challenges this demographic presents, there is a high need for a potential complementary or alternative diagnostic tool for early detection of cSDH.

This study is the first to prospectively evaluate the clinical utility of QP specifically in elderly patients with cSDH undergoing surgical evacuation. Although our finding—that QP did not reveal pathological NPI values—is consistent with established neuroanatomical principles and thus not inherently surprising (Chen et al., 2011; Jahns, 2019; Lussier et al., 2019; Meeker et al., 2005; Olson and Fishel, 2016), it provides important clinical quantification previously unavailable. Specifically, our data confirm explicitly that even substantial hematoma volumes can coexist with normal pupillary responses in elderly patients, due primarily to age-related cortical atrophy and enhanced intracranial compliance creating additional intracranial reserve space (Trotter, 1914; Yamashima and Friede, 1984; Lee, 2016; Familiari et al., 2023; Evans, 1942). Therefore, clinicians should not rely exclusively on pupillometry for early detection or triage decisions in this population, as normal pupillary responses do not necessarily exclude significant intracranial pathology. This insight contributes incrementally to refining diagnostic algorithms and highlights the need for complementary diagnostic modalities to accurately identify clinically significant hematomas in older patients.

To further elucidate this phenomenon, we specifically incorporated measurements to assess radiological hydrocephalus and cortical atrophy. Our data confirmed that older patients with cSDH often exhibit significantly reduced brain volumes and enlarged ventricles. These findings are in line with the etiopathogenetic model proposed by Familiari et al. (2023), who demonstrated that cortical atrophy is a key trigger for cSDH formation. In their extensive morphological and ultrastructural analyses, Familiari and colleagues highlighted that reduced brain volumes, as reflected by indexes like the RCA, play a central role in initiating a cascade of events: enlargement of subarachnoid spaces, minor transudation of CSF across membranes, neomembrane formation, neovascularization, and a chronic inflammatory milieu. Over time, these processes enable the gradual formation and expansion of cSDH without abrupt increases in ICP.

Interestingly, our results also showed a significant relationship between GCS and measures of brain atrophy. Contrary to the simplistic notion that severe clinical presentations (e.g., a lower GCS) directly result from subdural hematoma size or mass effect, our findings suggest a more intricate interplay.

In cases of larger hematomas, our findings suggest that higher volumes may begin to surpass the compensatory capacity of the atrophic brain, potentially affecting ICP and thus influencing pupillary reactivity. We observed a correlation between greater hematoma volume and subtle changes in NPI values, implying that QP could hold diagnostic value for a subset of cSDH patients presenting with very large hematomas. Although this trend did not achieve statistical significance, it underscores the need for further research to determine the specific volume thresholds and patient cohorts in which QP monitoring may become clinically meaningful.

### 4.1 Limitations

This study's conclusions must be interpreted within the context of several key limitations. First, although QP has shown potential as a non-invasive neurological monitoring modality, its clinical validity and utility in cSDH management, particularly in older patients, remain unproven. Our findings did not establish significant correlations between QP metrics and intracranial pressure elevations or changes in neurological status, suggesting that QP's sensitivity to subtle alterations may be limited or that its application in this specific population and clinical context is not fully optimized.

Second, the sample size (n = 26) was relatively small, restricting both the statistical power and the precision of our estimates. A formal sample-size calculation was performed post hoc using G∗Power 3.1, revealing that our cohort (n = 26) provided approximately 75 % statistical power to detect a moderate correlation (ρ = 0.5) at an alpha level of 0.05. Given the low annual volume of surgically treated, neurologically intact cSDH at our centre (≈30 cases), we prospectively framed this investigation as a single-centre feasibility pilot, anticipating subsequent multi-centre expansion. Although our institution manages a high volume of cSDH cases annually, quantitative pupillometry (QP) has only recently been implemented in our clinical practice. Therefore, this study represents our initial experience using QP prospectively in this specific patient cohort. Our intention was to present preliminary data to inform subsequent, larger-scale research.

Variations in hematoma volume and the advanced age of participants introduce additional complexity, as the interplay of these factors and baseline cerebral atrophy or impaired autoregulation is not well understood and may confound QP-based assessments. Third, the characterization of dementia and cortical atrophy relied predominantly on imaging and clinical assumptions rather than comprehensive neuropsychological testing or ancillary investigations (e.g., lumbar puncture, biomarker analysis). The absence of rigorously confirmed cognitive or neurodegenerative diagnoses may have masked relevant stratifications within the study population, influencing the interpretation of QP's potential role as a diagnostic or monitoring tool. Our study lacks a direct control group such as younger cSDH patients or patients with other conditions known to increase intracranial pressure (ICP). This limitation restricts the specificity of conclusions regarding QP's diagnostic value exclusively to elderly cSDH patients. Future research should include comparative analyses involving control groups to better define QP's diagnostic and prognostic roles. Our methodology did not incorporate direct ICP monitoring, primarily because invasive ICP measurements are uncommon in stable, elderly cSDH patients without acute neurological deterioration. Given the neuroanatomical context of elderly patients—characterized by increased intracranial compliance due to age-related brain atrophy—future studies might cautiously consider integrating minimally invasive or telemetric ICP-monitoring techniques to validate pupillometric findings directly against ICP data. This study did not include longitudinal follow-up data or formal assessments of long-term functional outcomes such as modified Rankin Scale (mRS) scores. Consequently, the relationship between initial QP findings and subsequent clinical recovery or neurological outcomes remains unexplored. In our study design we would incorporate structured follow-up assessments to determine whether early pupillometric measures could potentially serve as prognostic indicators of clinical recovery or risk of cSDH recurrence. Looking ahead, future research should involve larger, more diverse patient cohorts and incorporate systematic neurological, cognitive, and radiological assessments. Such studies might identify hematoma volumes, degrees of atrophy, or cognitive impairment thresholds beyond which QP measurements offer clinically meaningful insights. By refining patient selection criteria, enhancing diagnostic accuracy, and integrating QP with other emerging monitoring techniques, it may be possible to more clearly delineate the scenarios in which pupillometry can significantly inform the management of cSDH in older adults.

## 5 Conclusions

Collectively, our findings reinforce the complex interplay between age-related cortical atrophy, intracranial compliance, and cSDH pathophysiology. The unique intracranial environment of elderly patients, characterized by enlarged ventricles, diminished brain parenchymal volume, and reduced vascular integrity, fundamentally alters both the clinical presentation and the diagnostic landscape. Whereas cSDH formation in younger individuals often coincides with more overt ICP elevations and corresponding clinical signs, older patients may harbor large hematomas with minimal pupillometric or neurological disturbances until a critical threshold is reached. By demonstrating that QP remains largely unremarkable in most elderly cSDH cases our results highlight the nuanced relationship between structure and function. More importantly, they underscore that reliable diagnosis currently still rely on cCT imaging.

## Authors’ contributions

All authors contributed to the study conception and design. Material preparation, data collection and analysis were performed by Lukas Klein and Pavlina Lenga. The first draft of the manuscript was written by Lukas Klein and PL and SK commented on previous versions of the manuscript. All authors read and approved the final manuscript.

## Consent to participate

QP is incorporated into standard care in our setting, informed consent was deemed unnecessary.

## Ethics approval

This study was conducted in accordance with the Declaration of Helsinki and approved by the local ethics committee (S-788/2021).

## Consent for publication

No individual person's data were included in this study.

## Data material availability

The datasets generated during and/or analyzed during the current study are available from the corresponding author on reasonable request.

## Human and animal ethics

Not applicable.

## Funding

None.

## Competing interests

The authors have no relevant financial or non-financial interests to disclose.
